# Key Notes

**Published:** 1892-04

**Authors:** Samuel Swan

**Affiliations:** 13 West Thirty-eighth St.; New York


### KEY NOTES.

PROTAGON.—For brain-fag, and the mental and physical debility after intense intellectual study, and the same condition following *la grippe.* The condition of Mr. Edward M. Field, as described in the papers, is a simillimum—no doubt that he could be cured.

VARIOLINUM.—For a dry, hard cough, from constant itching or irritation in the larynx and trachea. Cough after *la grippe.*

MORBILIN.—Constant cough in children, from itching or irritation in larynx.

BUBOIN-SYPHILITICA.—For adenia (Hodgkin's disease), glandular diseases, syphilitic gummata.

TUBERCULINUM.—For a hard cough, resulting in the expectoration of yellow mucus, mixed with common mucus, or of a white lump, or a quantity of white substance, looking like thick milk, and especially if there is soreness in either or both lungs, when coughing or taking a deep breath. (Incipient tuberculosis).

MORBILIN.—For measles.

DIPHTHERINUM.—For diphtheria.

SCARLETINUM.—For scarlet fever, either of the smooth Sydenham variety, or for the eruptive.

VARIOLINUM.—For small-pox in all stages, and as a preventive.

PYROGEN.—For all stages of blood-poison, whether from injuries, operations, malignant diseases, or malaria.

SYPHILINUM.—In any disease where the aggravation begins after four P. M., *and ceases at daylight.*

LAC-FELINUM.—Pain in and through left side of head; pain through the left eye-ball into the brain; ciliary iritis.

SANTONINE.—Bright's disease, gout, rheumatism, and especially if there is an *intolerable, offensive odor from the urine.*

Magnetismus-Australis.—For ingrowing toe-nail; relieves the pain instantly, cures quickly and permanently.

*Cholesterine.*—Almost a specific for gall-stone colic; relieves the distress at once.

*Erysipelin.*—Has cured many cases of erysipelas.

*Eczematin-syphilitica.*—Is very effective, especially if prepared from the patient's disease.

The above remedies were given in the *highest* potencies, and the results verified by other physicians beside myself.

Samuel Swan, M. D.,
New York, March, 1892. 13 West Thirty-eighth St.

